# Evaluation of the short-term curative effect of closed reduction in the treatment of developmental dysplasia of the hip based on three-dimensional magnetic resonance imaging finite element analysis

**DOI:** 10.1186/s12891-022-05401-x

**Published:** 2022-05-14

**Authors:** Jiani Liu, Tianyang Gao, Jia Li, Hui Shan, Shinong Pan

**Affiliations:** 1 Department of Radiology, Cancer Hospital of China Medical University, Liaoning Cancer Hospital & Institute, Shenyang, China; 2 Department of Radiology, Shengjing Hospital of China Medical University, Shenyang, China; 3 School of Mechanical Engineering and Automation, Northeastern University, Shenyang, China

**Keywords:** Hip, Developmental dysplasia of the hip, Closed reduction, Magnetic resonance imaging, Finite element analysis

## Abstract

**Background:**

Based on the Digital Imaging and Communications in Medicine (DICOM) data of three-dimensional magnetic resonance imaging (3D-MRI), finite element models of the hip joints of children with developmental dysplasia of the hip were established. The primary objectives included simulation and analysis of the finite element model pre- and post-closed reduction under different stances and loads, and evaluation of the size and distribution of von Mises stress in the acetabulum and femoral head pre- and post-operation and the short-term effects.

**Methods:**

Acetabular index measurements of both the unaffected and affected sides were conducted, alongside International Hip Dysplasia Institute (IHDI) classification of the affected hip. Establishing the finite element model of both the affected and unaffected hips was based on the 3D-T1WI sequence DICOM data, using Mimics, 3-matic, and Ansys software, before and after closed reduction surgery. The size and distribution data of von Mises stress on the affected side of the acetabulum and femoral head were collected pre- and post-operation.

**Results:**

The study indicated that the increasing acetabular index of the affected hip was directly proportional to the increasing severity based on IHDI classification (*P* < 0.05). Preoperative IHDI classification significantly correlated with the von Mises stress (*r* = 0.560–0.569, 0.562–0.564, *P* < 0.05). Under different stances and load conditions, the von Mises stress on the affected side post-operation was lower than that noted pre-operation (*P* < 0.01), while that on the acetabulum increased proportionally to the load. Although the magnitude and distribution of von Mises stress on the affected side of the acetabulum were similar to those on the healthy side post-operation, there were statistical differences between the two (*P* < 0.01). The von Mises stress of the lateral column of the femoral head post-operation was significantly lower than that noted pre-operation (*P* < 0.01). While the high-stress points of the lateral column disappeared post-operation, the von Mises stress was evenly distributed in the femoral head.

**Conclusions:**

The 3D-MRI finite element could provide the von Mises stress value and distribution characteristics of the acetabulum and femoral head pre- and post-operation. Closed reduction can, therefore, improve the size and distribution of von Mises stress on the affected acetabulum and femoral head.

## Background

Developmental dysplasia of the hip (DDH) is one of the most common hip diseases in children. DDH includes acetabular dysplasia, hip semi-dislocation, and hip dislocation [1]. Treatment methods vary depending on the age of children with DDH. Currently, closed reduction (CR) and spica cast application are standard treatments for 6–18-month-old children with DDH [2, 3]. The treatment aims at restoring the concentric circle position of the femoral head and acetabulum and maintaining joint stability, thus stimulating the normal development of the hip and ensuring minimal damage to the femoral head. Unfortunately, avascular necrosis (AVN) of the femoral head remains one of the most severe complications after closed reduction [4–10].

Finite element analysis (FEA) is a dispersion method in numerical calculations, comprising the development and application of matrix methods in the fields of structural mechanics and elastic mechanics. FEA replaces biomechanical experiments to a certain extent and can control experimental conditions and simulate the biomechanical conditions of the human body. In the biomechanical evaluation, according to the fourth strength theory, no matter what stress state, the main distortion energy density reaches a certain limit value related to the material properties, and the material will yield. Similarly, bone is prone to yield failure. At this time, Von Mises Stress can be used as the failure judgment index. Following are the basic steps for FEA: image scan, geometric model reconstruction, division finite element mesh, skeletal material attribute definition, determination of boundary conditions, load loading, and final analysis. The earliest use of 2-dimensional (2D) FEA was by Andriacchi et al. [11], who studied the image factors of early femoral fat fatigue fractures after total hip replacement. Due to the simple modeling involved in the 2D-FEA method, construct complex morphology and multiple material composite model processes have relatively high error rates, alongside image overlapping distortion. With continuous improvement and expansion of three-dimensional (3D)-FEA technology, the characteristic parameters and geometric details can be automatically extracted from the magnetic resonance imaging (MRI)/computed tomography (CT) data. By changing any parameter, multiple clinical-pathological states are simulated, biological changes of the hip physiology and pathological processes are better analyzed, and clinical individualization treatments are guided.

It has been reported that the 3D-FEA simulates the absence of different DDH closed reduction outgoing abductions, observes the changes of von Mises stress in the acetabular and femoral head of the hip joint, explores the possible mechanism of AVN after closed reduction, and provides mechanical guidance for clinical surgery [12, 13]. This study aimed to establish a 3D-MRI FEA model before and after closed reduction surgery. This includes simulation analysis of different loads and station posture, and evaluation of the von Mises stress change between the acetabulum and femoral head as well as the short-term efficacy after closed reduction.

## Materials and methods

### Clinical information

The medical records of DDH children in Shengjing Hospital of China Medical University from December 2019 to December 2020 were collected. All children received closed reduction treatment, and the general clinical data and MRI data were recorded. The hospital ethics committee approved this study (approval no. 2020PS754K).

The inclusion criteria were as follows: 1) the age at hospitalization < 18 months; 2) closed reduction treatment under general anesthesia; 3) single side DDH; 4) MRI review 1 week after surgery. The exclusion criteria were as follows: 1) surgical treatment with reset failed; 2) DDH accompanied with AVN; 3) neuromuscular DDH; 4) incomplete imaging data (lack of preoperative or postoperative MRI examination); 5) contraindications for MRI examination; 6) no treatment or failure of Pavlik treatment before surgery..

This study included 15 children (30 hips), including 3 boys (6 hips) and 12 girls (24 hips); 9 cases of the hip on the left side and 6 cases of the hip on the right side. The preoperative age of children was 4–18 months and the median age was 9 months. Body weight was 8–15 kg, with an average weight of 10.22 ± 1.91 kg.

### Scanning method

Before Pelvic MRI examination, all children were sedated and hypnotized by enema with chloral hydrate(0.05 g/kg) and physiological saline(10 mL). All MR images were collected with 3.0 T magnetic resonance (Philips Ingenia CX 3.0 T, Netherlands) and scanning with body launching and accepting coil. Conventional MRI sequences (coronal T1WI, T2WI, PDWI, transverse T2WI, and sagittal PDWI) and transverse 3D-T1WI were scanned from the anterior superior iliac spine to the center of the femoral shaft as the reference plane, and the head in supine position was advanced with keep the legs horizontal (Table 1). The scanning time was approximately 8–10 min.

**Table 1:** Magnetic resonance imaging parameters

	Cor-T1WI	Cor-T2WI	Cor-T2WI-FS	Tra-T2WI	Sag-T2WI-FS	3D-T1WI
TR (ms)	482.06	3228.26	2468.96	3475.28	2442.70	400
TE (ms)	18	130	50	110	55	27
FOV(mm)	376 × 376	376 × 376	376 × 376	360 × 360	250 × 250	370 × 370
Section thickness (mm)	3.5	3.5	3.5	3.5	7	1.16

*TR*  Repetition time, *TE*  Echo time, *FOV*  Field of view, *FS * Fat suppression

### Imaging analysis

The horizontal line of the 2 triangular hyaline cartilage is designated as the H-line; the connection between the acetabular upper edge and the lower lateral point of the acetabular iliac bone is Acetabular index (AI) with the H line. The International Hip Dysplasia Institute (IHDI) classification standard [14] is used to classify DDH into hip dislocation degrees before surgery. Postoperative femoral head necrosis and classification were performed according to the Salter criteria [15] and Kalamchi–MacEwen classification [16, 17]. PACS image reading system (Neusoft, Shenyang) was used to measure the image parameters of all MRI images.

### Finite element model establishment and analysis

#### Establishment of the geometric model of the affected hip and meshing

**Fig. 1:**
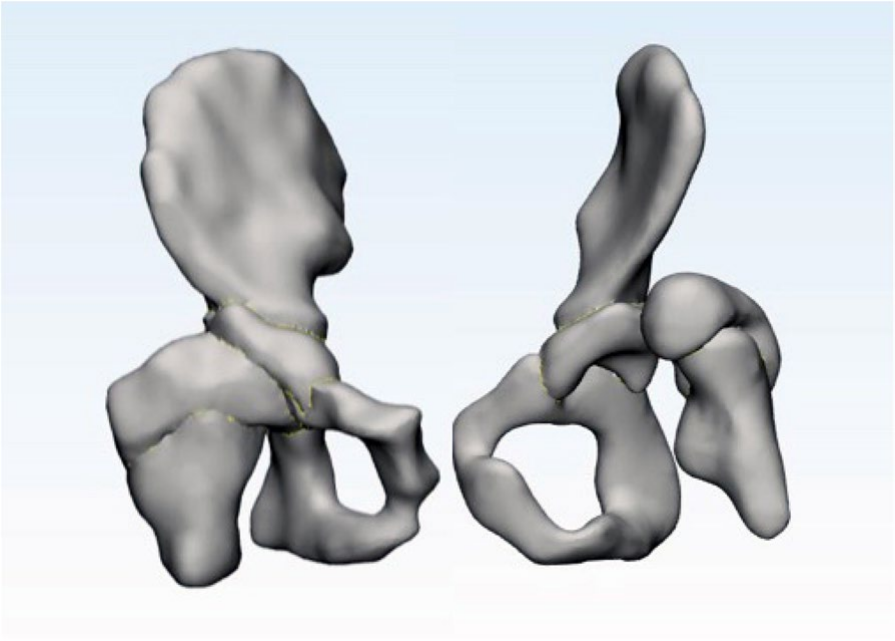
Three-dimensional model of the hip

The Digital Imaging and Communications in Medicine (DICOM) data of the affected hip 3D-T1WI were imported into the Mimics Research 21.0 software (Materialise, Belgium). The iliac crest, pubic bone, ischium, femoral head, and superior femoral bone, as well as the per femoral head and acetabular cartilage, were modeled by using magnetic lasso, regional growth, mask editing, and 3D model calculation. Import the 3D model into 3-material research 13.0 software (Belgium materialise) for mesh optimization to generate surface network. The optimized surface mesh is used to generate tetrahedral mesh, which is imported into FEA module, and the three-dimensional finite element model is output to ANSYS software in CDB format. In ANSYS 19.0 software, and the proximal femur was subjected to grid densification. A total of 186,351 units were generated, including 116,849 units of cortical bone and 69,502 units of cancellous bone (Fig. 1). Referring to the previous literature, the grid is assigned with different material properties to the imported model.

#### Model material assignment

According to Zhenmin Zou[18] and Jong-eunkim [19], the material properties of children's hip joints are studied, and each material is assigned. The acetabulum and femoral cartilage are molded into homogeneous, isotropic and linear elastic materials, where Young's modulus E = 15 MPa, Poisson's ratio ν = 0.45. The cortical bones of the pelvis and femur are homogeneous and isotropic, with the Young's modulus, E = 17 MPa and Poisson's ratio, ν = 0.3. The pelvis and femur cancellous bones are homogeneous and isotropic, with the Young's modulus, E = 70 MPa and Poisson's ratio, V = 0.3. The joints are connected by contact elements and constrained by friction coefficient. When the gap between acetabulum and cartilage is less than a certain value, the interaction will occur, and the contact type is face-to-face contact. In this study, the friction coefficient between the two contact interfaces is defined as 0.4 and the contact gap is defined as 0.05 mm.

#### Boundary restraint and load application

Referring to the reported boundary conditions, all displacements of the sacroiliac side, pubic symphysis, and distal femur were constrained, and the displacements and rotations in all directions were zero [20]. The contact between the femoral proximal end and the acetabular cartilage is defined as the contactless surface. In the simulation of a 2-legged posture, since the lower limbs account for 1/3rd of the total mass, vertical downward gravity is applied to the upper surface of the iliac bone, which is about 2/3rd of the total mass. In the simulation of single-legged standing posture, the single lower limb accounts for 1/6th of the total mass, so vertical downward gravity is applied on the upper surface of the iliac bone, which is about 5/6th of the total mass [21, 22]. Based on Herring's three-pillar view of Legg-Perthes disease [23], the femoral head was divided into the outer column, central column, and medial column for analysis. The acetabulum and femoral head von Mises stress on the affected side before the operation, after the operation, and on the healthy side under 1–3 times body weight, were measured respectively in the single- and double-legged standing postures.

### Statistical analyses

SPSS 21.0 statistical software was used for data analysis, and the measurement data are presented as $\overline{x }$±*s*. One-way analysis of variance (ANOVA) was used to compare the differences in hip AI between different IHDI classifications. The paired-sample t-test was used to compare (1) the von Mises stress of the iliac bone and femoral cartilage before and after the operation, (2) the von Mises stress of the iliac bone and femoral cartilage after the operation, and (3) the contralateral side. Pearson correlation analysis was used to analyze the 2-variable correlation between the preoperative IHDI classification and the von Mises stress of the acetabulum and femoral head. *P* < 0.05 indicates that the difference is statistically significant.

## Results

### Imaging analysis

The AI values of all affected hips were 42.50° ± 3.52°, with a range of 34.43°-48.00°. The healthy hip AI value was 29.14° ± 3.66°, and the range was 23.57°-34.72°. According to IHDI classification, there were 3 cases (20%) of II type, 8 cases (53.3%) of III type, and 4 cases (26.7%) of IV type. The AI of affected hip increased proportionately to the increase of IHDI type (*P* < 0.05), and the II-IV type AI values were (37.60 ± 2.78)°, (42.37 ± 1.69)°, and (46.45 ± 1.10)°, respectively. Preoperative IHDI typing was significantly associated with acetabulum and femoral head von Mises stress on the affected side (Table 2). The von Mises stress of the acetabulum and femoral head on the affected side increased proportionately to the increase of IHDI classification when 1–3 times the body weight was applied in the single-legged and the double-legged standing posture, respectively. Postoperative 3D MRI showed that the concentric acetabulum and femoral head between the affected acetabulum and the femoral head were restored, the joint gap was equipped, and the Shenton line was continuous. No re-dislocation or AVN occurred in 15 affected hip joints.

**Table 2:** The correlation between preoperative IHDI classification and von Mises stress of the affected acetabulum and femoral head

r	Single-leg standing			2-leg standing		
	1 × body weight	2 × body weight	3 × body weight	1 × body weight	2 × body weight	3 × body weight
acetabulum	0.563	0.653	0.560	0.569	0.565	0.563
femoral head	0.563	0.562	0.562	0.563	0.564	0.562

### von Mises stress and distribution of acetabulum

The results of von Mises stress of the acetabulum on the affected side when 1–3 times the body weight was applied to the single and double-legged standing positions are shown in Table 3. Regardless of single- or double-legged standing positions, the von Mises stress of the affected hip post-operation was lower than the value pre-operation. Further, the acetabular von Mises stress increased with the increase of load (Table 3).

**Table 3:** The von Mises stress of the acetabulum before and after the operation under different loads

von Mises	Single-leg standing (MPa)				2-leg standing (MPa)			
	preoperative	postoperative	*t*	*p*	preoperative	postoperative	*t*	*p*
1 × body weight	0.95 ± 0.18	0.54 ± 0.10	20.667	< 0.01	0.68 ± 0.13	0.42 ± 0.08	20.817	< 0.01
2 × body weight	1.83 ± 0.34	1.07 ± 0.20	20.978	< 0.01	1.50 ± 0.28	0.91 ± 0.17	20.786	< 0.01
3 × body weight	2.71 ± 0.51	1.60 ± 0.30	20.544	< 0.01	2.17 ± 0.41	1.30 ± 0.24	20.533	< 0.01

Although the magnitude of von Mises stress on the affected side was similar to the value observed on the uninjured side (Table 4), there was statistical significance between the two (*P* < 0.01). This is evident in the acetabular stress cloud map, alongside the presence of a high-stress concentration point outside the upper boundary of the acetabulum, which increases as the load increases.

**Table 4:** The von Mises stress of the acetabulum on the unaffected side and the operation under different loads

von Mises	Single-leg standing (MPa)				Double-leg standing (MPa)			
	unaffected	postoperative	*t*	*p*	unaffected	postoperative	*t*	*p*
1 × body weight	0.59 ± 0.11	0.54 ± 0.10	22.205	< 0.01	0.46 ± 0.09	0.42 ± 0.08	19.071	< 0.01
2 × body weight	1.14 ± 0.21	1.07 ± 0.20	20.949	< 0.01	0.93 ± 0.17	0.91 ± 0.17	15.332	< 0.01
3 × body weight	1.69 ± 0.32	1.60 ± 0.30	18.445	< 0.01	1.30 ± 0.24	1.36 ± 0.25	18.564	< 0.01

**Fig. 2:**
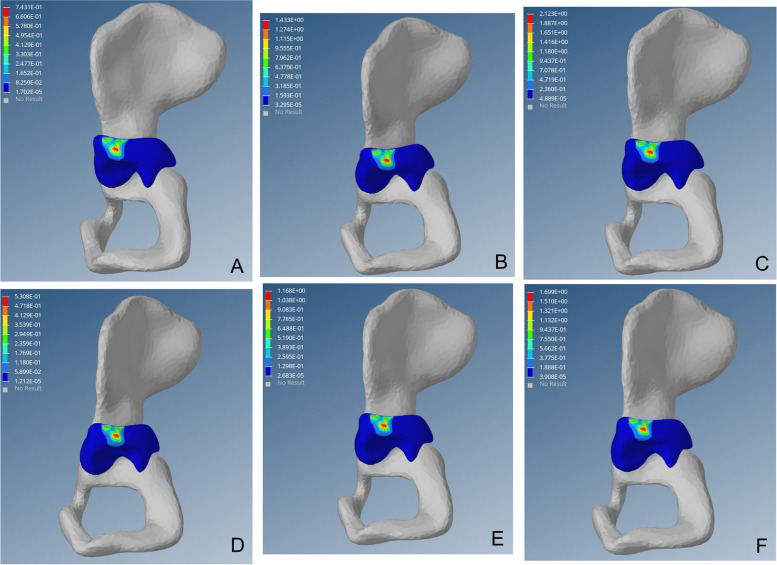
Preoperative von Mises stress distribution on the affected side of the acetabulum. Fig (A-C) von Mises stress cloud diagram of the acetabulum under a load of 1–3 times the body weight when standing on one leg. Fig (D-F) von Mises stress cloud diagram of the acetabulum under a load of 1–3 times the body weight when standing on both legs

**Fig. 3:**
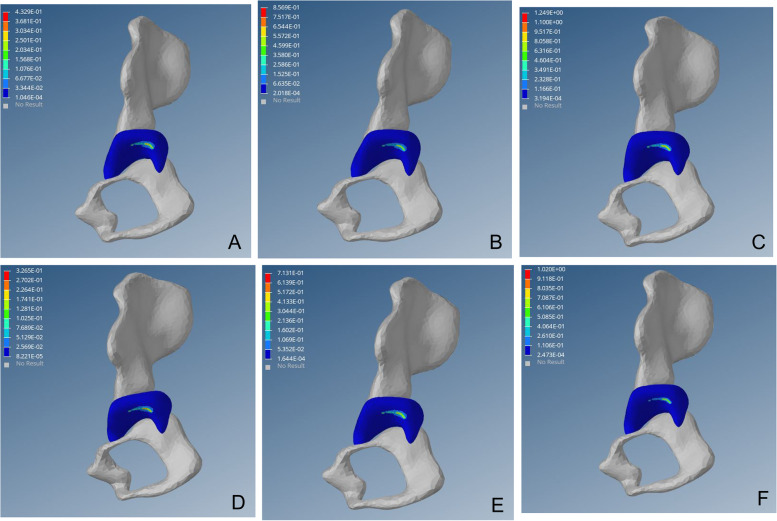
von Mises stress distribution on the affected side of the acetabulum post-operation. Fig (A-C) von Mises stress cloud diagram of the acetabulum under a load of 1–3 times the body weight when standing on one leg. Fig (D-F) von Mises stress cloud diagram of the acetabulum under a load of 1–3 times the body weight when standing on both legs

**Fig. 4:**
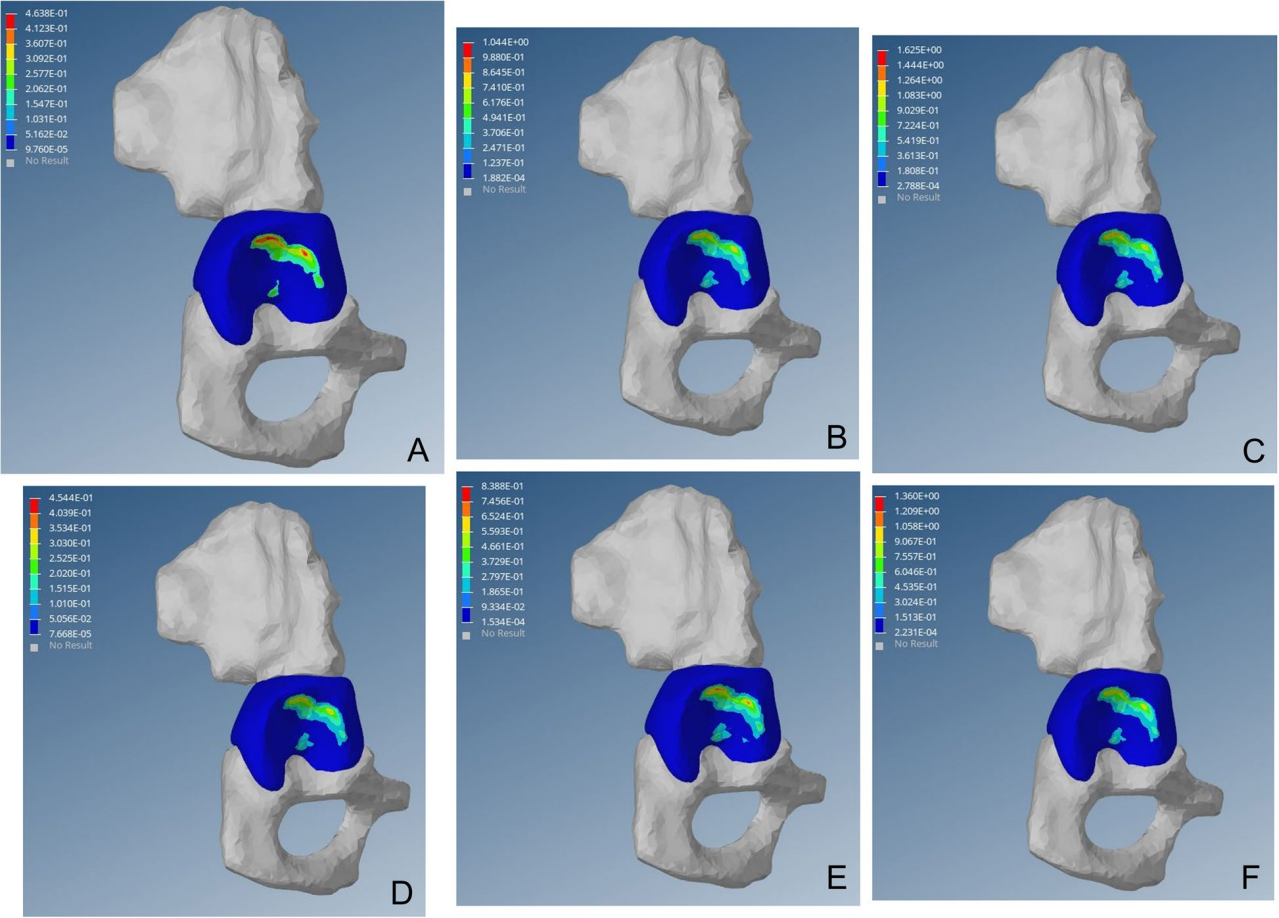
von Mises stress distribution in the healthy side of the acetabulum. Fig (A-C) von Mises stress cloud diagram of the acetabulum under a load of 1–3 times the body weight when standing on one leg. Fig (D-F) von Mises stress cloud diagram of the acetabulum under a load of 1–3 times the body weight when standing on both legs

Following operation, the high-stress point disappeared at the lateral edge of the upper region of acetabulum and von Mises stress distributed evenly inside the acetabulum. The stress was transmitted from the central upper region of the acetabulum to an inward and downward progression and gradually weakened radially to the periphery. This was similar to the distribution and transmission of stress on the healthy hip (Figs. 2, 3 and 4).

### Stress and distribution of femoral head von Mises

The stress of the outer column of the femoral head is higher than that of the central and inner columns. The stress of the outer column of the femoral head after surgery was significantly less than that observed preoperatively (Table 5). Although the von Mises stress of the femoral head on the affected side was similar to that on the healthy side post-operation (Table 6), there was no statistical significance in von Mises stress of the femoral head under the load of 3 times body weight in the single-legged posture or 1 time the body weight applied in the double-legged posture. Nonetheless, statistical significance was observed in all remaining parameters (*p* < 0.01).

**Table 5:** von Mises stress of the femoral head before and after operation under different loads

von Mises	Single-leg standing (MPa)					2-leg standing (MPa)		
	preoperative	Postoperative	*t*	*P*	preoperative	postoperative	*t*	*p*
1 × body weight	1.06 ± 0.20	0.59 ± 0.11	20.149	< 0.01	0.75 ± 0.14	0.49 ± 0.09	20.032	< 0.01
2 × body weight	2.04 ± 0.38	1.13 ± 0.21	20.666	< 0.01	1.66 ± 0.31	0.97 ± 0.18	20.632	< 0.01
3 × body weight	3.02 ± 0.56	1.80 ± 0.34	20.616	< 0.01	2.41 ± 0.45	1.38 ± 0.26	20.855	< 0.01

**Table 6:** The von Mises stress of the femoral head on the unaffected side and post operation under different loads

von Mises	Single leg standing (MPa)				Double-leg standing (MPa)			
	unaffected	Postoperative	*t*	*p*	unaffected	postoperative	*t*	*p*
1 × body weight	0.57 ± 0.11	0.59 ± 0.11	10.693	< 0.01	0.49 ± 0.10	0.49 ± 0.09	1.848	> 0.05
2 × body weight	1.15 ± 0.22	1.13 ± 0.21	15.332	< 0.01	0.98 ± 0.18	0.97 ± 0.18	12.475	< 0.01
3 × body weight	1.81 ± 0.34	1.80 ± 0.34	1.640	> 0.05	1.41 ± 0.26	1.38 ± 0.26	18.500	< 0.01

**Fig. 5:**
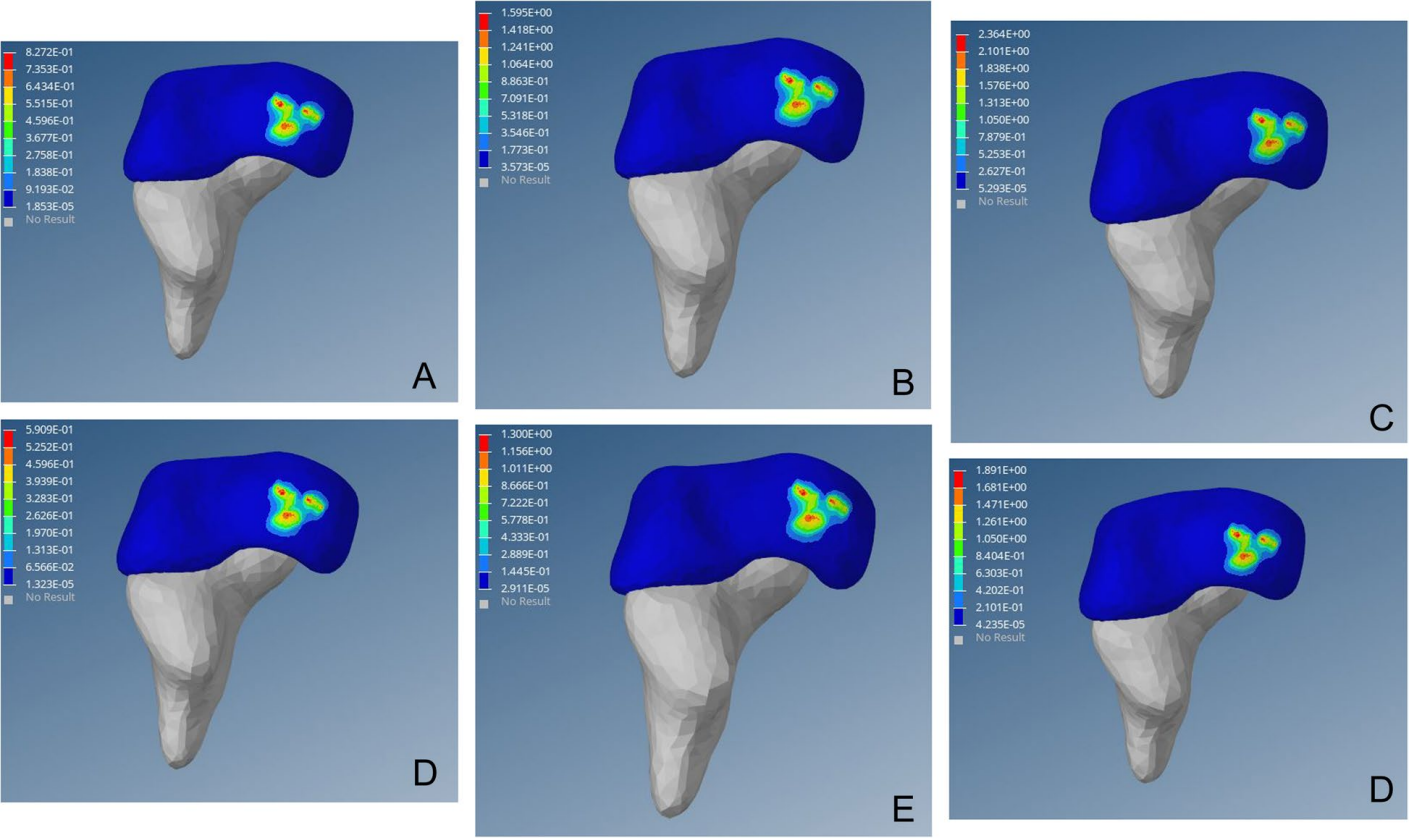
von Mises stress distribution of the affected femoral head before operation. Fig(A-C) von Mises stress cloud diagram of the femoral head under a load of 1–3 times the body weight when standing on a single leg. Fig (D-F) von Mises stress cloud diagram of the femoral head under a load of 1–3 times the body weight when standing on both legs

**Fig. 6:**
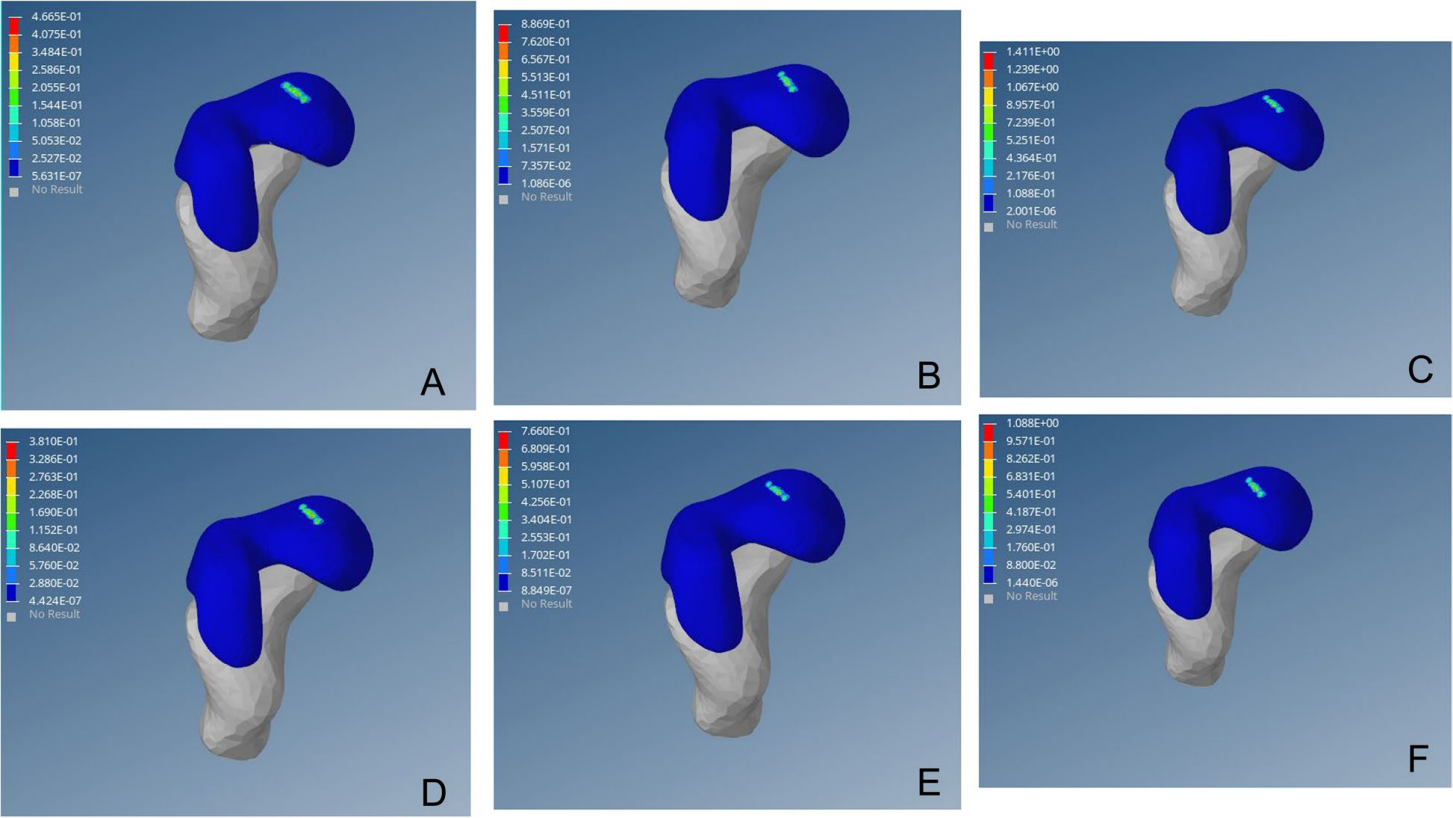
von Mises stress distribution of the affected femoral head after surgery. Fig (A-C) von Mises stress cloud diagram of the femoral head under a load of 1–3 times the body weight when standing on a single leg. Fig (D-F) von Mises stress cloud diagram of the femoral head under a load of 1–3 times the body weight when standing on both legs

**Fig. 7:**
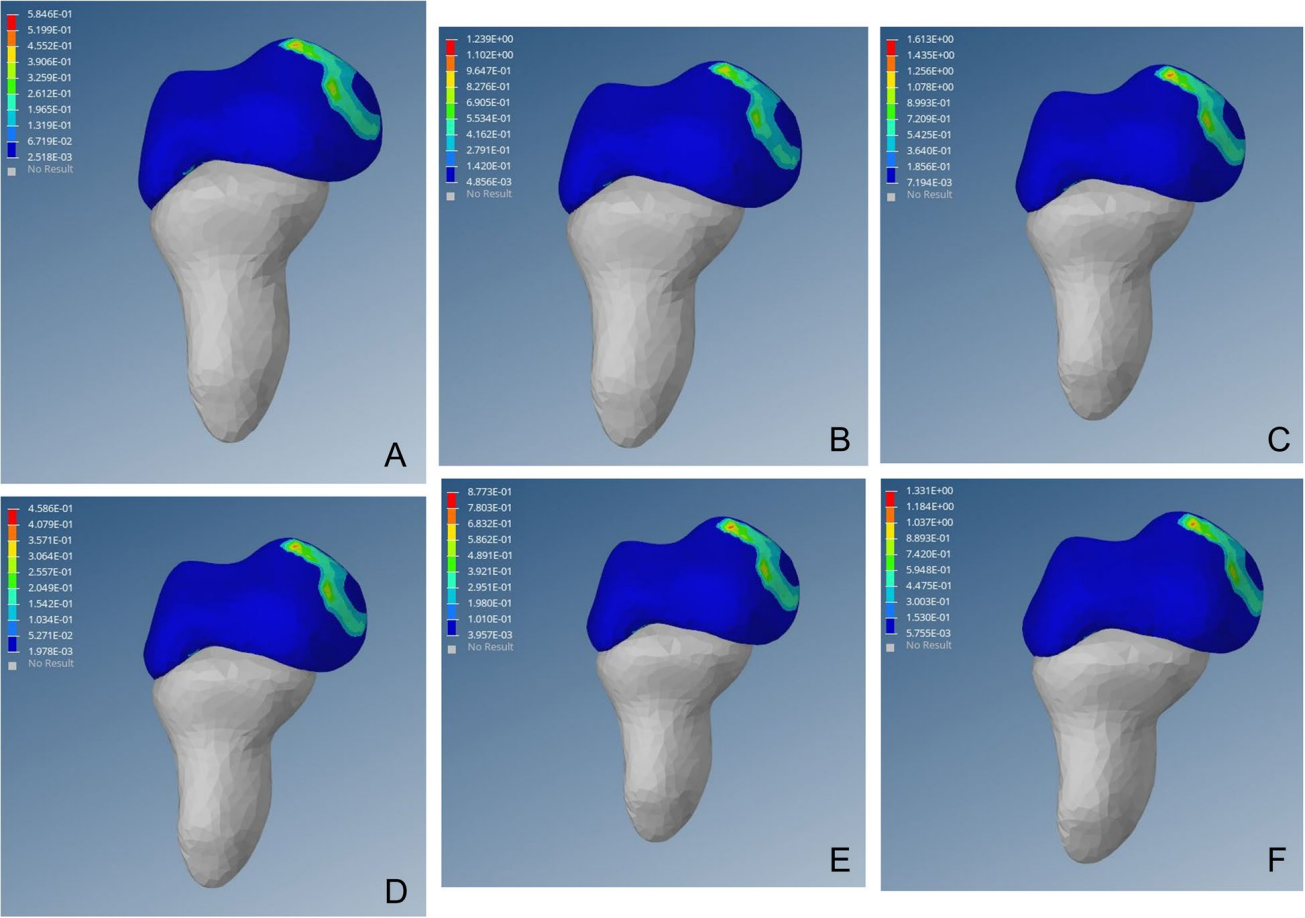
von Mises stress distribution of the healthy side femoral head. Fig (A-C) von Mises stress cloud diagram of the femoral head under a load of 1–3 times the body weight when standing on a single leg. Fig (D-F) von Mises stress cloud diagram of the femoral head under a load of 1–3 times the body weight when standing on both legs

As shown in the femoral head stress cloud map, the outer strip of the preoperative femoral head appeared at a high-stress point, which corresponds to the top outer edge of the acetabulum and increased proportionately to the load. After surgery, the high-stress point of the outer column disappeared, and the femoral head Von Mises stress distributed evenly and radiated to all sides, similar to the scientific side (Figs. 5, 6 and 7).

## Discussion

To restore the containing structure of the femoral head and acetabulum, the treatment is usually based on morphology (restoration of normal radiological geometrical parameters) and biomechanics [24–29]. In imaging measurement systems [30, 31], IHDI classification correlated with the pathological morphology of DDH, where the affected hip AI increased with the increased severity of IHDI classification [32]. AI, as a classic indicator to evaluate the effect of closed reduction [33], reflects the inclination of the joint stress surface and the inclusion degree of the femoral head and acetabulum [34]. The IHDI division is directly proportional to the AI, and the higher they are, the worse the inner inclusiveness, resulting in a smaller contact area of the head hone, a larger stress in the unit area, worsened joint stability, and greater femoral head stress. In this study, the preoperative AI of all affected hips was higher than that of the healthy side and increased with the increased severity of the IHDI type. The acetabulum and femoral head von Mises stress on the affected side were higher than those on the healthy side when 1–3 times of body weight was applied in the single and double standing positions. The preoperative IHDI classification was significantly and proportionately correlated with the von Mises stress of the acetabulum and femoral head on the affected side.

Wolff Theory indicates that bone growth is closely related to stress, and high-strength load can inhibit osteocyte activity, promote osteogenic cells, and inhibit bone growth. In addition to the literature [13, 35, 36], in a dysplastic hip, the rate of inner growth of the femoral proxima is high, causing an increase in neck angle and subsequent hip varus. In this study, under the single-legged posture and legs station posture, an abnormal high-stress distribution under the outer column of the femoral head was noted (Fig. 5), and with the increasing load and von Mises stress, these abnormalities are likely to inhibit the growth of the proximal end of the femoral end. Moreover, the innermost rapid growth may result in femoral collo-diaphyseal angle increased and hip varus. After closed reduction, the abnormally high-stress distribution of the outer column of the proximal femur disappeared, the load stress was evenly distributed in the femoral head and transmitted to the 4-week radiation, which was similar to the aerial femoral head (Figs. 6 and 7).

Von Mises stress of the femoral head reflected the mechanism of load transmission and mechanical stress state in the joint. Previous finite element studies [12, 13, 37–40] have shown that the von Mises stress of the femoral head increased alongside abduction angle. Further, high von Mises stress could lead to the compression of the blood vessels of the femoral head to the joint cavity, as well as cartilage injury, osteoarthritis (OA), and even the occurrence of AVN. Vafaeian et al. [27, 28] simulated the Pavlik sling treatment, a finite element model, to study von Mises stress on AVN. They found that with extreme leg abduction angles, the acetabular front and rear wall and the corresponding femoral head outside von Mises stress increased and could cause AVN.

At the same time, the outer column of the femoral head is the main carrier area of the femoral head, but it is in primary contact with the acetabulum. Furthermore, when the necrotic lesion involves the outer column, its mechanical carrying capacity is poor [41–43]. Wen et al. [44, 45] found that when the necrotic lesion involves the outer column (especially extremely external or the total femoral head type), the elastic modulus and yield strength of bone tissue of necrosis are below those seen with normal tissue. The outer column is also a stress-centralized region. When the von Mises stress index is higher than the critical value, the local microfracture is easier to increase, and even risks femoral head collapse.

In this study, the abnormally high von Mises stress distribution of the lateral femoral head column was observed in all 15 patients before surgery. With increasing load, the von Mises stress of the femoral head outer column also increased, which may lead to increased pressure inside the femur and compression of the nourishing artery, thus increasing the risk of AVN and collapse of the femoral head. This has guiding significance for clinical treatment. In the stress cloud map of the femoral head after surgery, the abnormally high von Mises stress before surgery disappeared in the outer column of the femoral head, and the distribution of von Mises stress in the 3 columns of the femoral head was uniform and gradually decreased radially to the periphery. Whether in the single-legged or double-legged posture, as the load increased, there was no high-stress point in the outer column of the femoral head. Therefore, closed reduction can reduce the risk of AVN and femoral head collapse.

DDH acetabular shape is shallow, resulting in insufficient coverage of the femoral head; long-term low coverage can lead to chronic overload of joint cartilage, resulting in cartridge damage, eventually leading to OA. Closed reduction, via restoration of the concentricity between the acetabulum and the femoral head, reduces the stress on the cartilage at the outer edge of the acetabulum, thereby preventing OA [46–48]. In this study, high-stress distribution was observed at the posterior upper edge of the acetabulum on the affected side before surgery. With the continuous increase of load, von Mises stress increased linearly, which may cause cartilage injury of the posterior upper edge and increase the risk of OA (Fig. 2). Postoperative acetabular stress peaks decreased from approximately 1/3 to 1/2. The postoperative acetabular stress cloud map did not show a high-stress concentration point on the posterior upper edge of the acetabulum, which was mainly distributed in the acetabular apex area, and the pressure gradually decreased radially to the periphery (Fig. 3). The imaging findings showed no OA-related manifestations, which was consistent with the studies of Abraham [49] and Knight [50].

This study has the following limitations and insufficiencies. First, this study may have a risk of bias as the sample number was relatively small, and the follow-up time was short. Therefore, it is not possible to evaluate the long-term effects on the hip comprehensively and completely, post-operation. Second, the load and boundary setting conditions cannot be fully in accordance with the real situation, and the finite element model of this study is relatively simplified, as it ignores the effect of the ligament around the hip joint following loading. In addition, this study has the problem of simple modeling of cancellous bone material in the process of model establishment, so there are some limitations in the process of stress evaluation. Moreover, because the material of the model is different from that of the entity, and the biological differences of individuals are not considered, this is also the problem of other finite element models. Finally, due to the existence of triangular hyaline cartilage in infants, the MRI image data when measuring bone pressure and supine position alone do not fully reflect the stress level of pelvis, and Drucker Prager equivalent stress can be used to evaluate the stress level of bone and cartilage in further research.

## Conclusion

In this study, the FEA method was used to compare the magnitude and distribution characteristics of von Mises stress on the acetabulum and femoral head before and after the closed reduction operation. After the operation, the high-stress points on the lateral edge of the top of the acetabulum and the lateral column of the femoral head disappeared, and their stresses were uniformly distributed in a radial pattern, which gradually weakened to the periphery. The magnitude and distribution of stress achieved on the affected hip was similar to that on the healthy side. Therefore, closed reduction can improve the size and distribution of von Mises stress on the affected acetabulum and femoral head.

## Data Availability

The datasets generated during the current study are not publicly available due the data is confidential but are available from the corresponding author on reasonable request.

## Abbreviations

3D-MRI: Three-dimensional magnetic resonance imaging
IHDI: International Hip Dysplasia Institute
DDH: Developmental dysplasia of the hip
CR: Closed reduction
AVN: Avascular necrosis
FEA: Finite element analysis
MRI: Magnetic resonance imaging
CT: Computed tomography
AI: Acetabular index
ANOVA: One-way analysis of variance
OA: Osteoarthritis

## References

[1] Weinstein SL, Mubarak SJ, Wenger DR (2004). Developmental hip dysplasia and dislocation: Part I. Instr Course Lect.

[2] Wang X, Peng J, Li D, Zhang L, Wang H, Jiang L, Chen X (2016). Does the optimal position of the acetabular fragment should be within the radiological normal range for all developmental dysplasia of the hip? A patient-specific finite element analysis. J Orthop Surg Res.

[3] Kotlarsky P, Haber R, Bialik V, Eidelman M (2015). Developmental dysplasia of the hip: What has changed in the last 20 years?. World J Orthop.

[4] Niziol R, Elvey M, Protopapa E, Roposch A (2017). Association between the ossific nucleus and osteonecrosis in treating developmental dysplasia of the Hip: updated meta-analysis. BMC Musculoskelet Disord.

[5] Liu YH, Xu HW, Li YQ (2019). Effect of abduction on avascular necrosis of the femoral epiphysis in patients with late-detected developmental dysplasia of the hip treated by closed reduction: a MRI study of 59 hips. J Child Orthop.

[6] Terjesen T, Horn J, Gunderson RB (2014). Fifty-year follow-up of late-detected hip dislocation: clinical and radiographic outcomes for seventy-one patients treated with traction to obtain gradual closed reduction. J Bone Joint Surg Am.

[7] Chen C, Doyle S, Green D, Blanco J (2017). Presence of the Ossific Nucleus and Risk of Osteonecrosis in the Treatment of Developmental Dysplasia of the Hip: A Meta-Analysis of Cohort and Case-Control Studies. J Bone Joint Surg Am.

[8] Vandergugten S, Traore SY, Docquier PL (2016). Risk factors for additional surgery after closed reduction of hip developmental dislocation. Acta Orthop Belg.

[9] Schur MD, Lee C, Arkader A (2016). Risk factors for avascular necrosis after closed reduction for developmental dysplasia of the hip. J Child Orthop.

[10] Li Y, Liu H, Guo Y, Xu H, Xun F, Liu Y, Yuan Z, Li J, Pereira B, Canavese F, Chinese Multicenter Pediatric Orthopaedic Study Group (CMPOS) (2020). Variables influencing the pelvic radiological evaluation in children with developmental dysplasia of the hip managed by closed reduction: a multicentre investigation. Int Orthop..

[11] Andriacchi TP, Galante JO, Belytschko TB, Hampton S (1976). A stress analysis of the femoral stem in total hip prostheses. J Bone Joint Surg Am.

[12] Yi Y, Jianlin L, Lei T (2017). Pathogenetic mechanism of avascular necrosis after dosed reduction of DDH: a research using digital human model. Orthopedic Journal of China.

[13] Zhang Z, Sui D, Qin H, Li H, Zhang Z (2020). Contact pressure distribution of the hip joint during closed reduction of developmental dysplasia of the hip: a patient-specific finite element analysis. BMC Musculoskelet Disord.

[14] Narayanan U, Mulpuri K, Sankar WN (2015). Reliability of a new radiographic classification for developmental dysplasia of the hip. J Pediatr Orthop..

[15] Salter RB, Kostuik J, Dallas S (1969). Avascular necrosis of the femoral head as a complication of treatment for congenital dislocation of the hip in young children: a clinical and experimental investigation. Can J Surg.

[16] A K, G D M. Avascular necrosis following treatment of congenital dislocation of the hip. J Bone Joint Surg Am. 1981:876–88. 7430175

[17] Bowen JR, Kruse R. Complication in the treatment of developmental dysplasia of the hip. In Complications in Pediatric Orthopaedic Surgery, 3rd ed, edited by Epps CH Jr, Bowen JR, 337-61. Philadelphia: JB Lippincott; 1994.

[18] Zou Z, Chávez-Arreola A, Mandal P (2013). Optimization of the position of the acetabulum in a ganz periacetabular osteotomy by finite element analysis. J Orthop Res.

[19] Kim JE, Li Z, Ito Y (2009). Finite element model development of a child pelvis with optimization-based material identification. J Biomech.

[20] Park SJ, Lee SJ, Chen WM (2018). Computer-assisted optimization of the acetabular rotation in periacetabular osteotomy using patient's anatomy-specific finite element analysis. Appl Bionics Biomech.

[21] Chao Xu, Yabo Y, Jingjing Ba (2016). Optimization of the center-edge angle of the acetabulum in a dega pelvic osteotomy by finite element analysis. Progress in Modern Biomedicine.

[22] Zhao X, Chosa E, Totoribe K (2010). Effect of periacetabular osteotomy for acetabular dysplasia clarified by three-dimensional finite element analysis. J Arthroplasty.

[23] Herring JA, Kim HT, Browne R (2004). Legg-Calve-Perthes disease - Part I: Classification of radiographs with use of the modified lateral pillar and Stulberg classifications. J Bone Joint Surg.

[24] Wang X, Peng J, LI (2016). Does the optimal position of the acetabular fragment should be within the radiological normal range for all developmental dysplasia of the hip? A patient-specific finite element analysis. J Orthop Surg Res.

[25] Chegini S, Beck M, Ferguson SJ (2009). The effects of impingement and dysplasia on stress distributions in the hip joint during sitting and walking: a finite element analysis. J Orthop Surg Res.

[26] Ike H, Inaba Y, Kobayashi N (2015). Effects of rotational acetabular osteotomy on the mechanical stress within the hip joint in patients with developmental dysplasia of the hip: a subject-specific finite element analysis. Bone Joint J.

[27] Vafaeian B, Zonoobi D, Mabee M (2017). Finite element analysis of mechanical behavior of human dysplastic hip joints: a systematic review. Osteoarthr Cartil..

[28] Vafaeian B, Adeeb S, El-Rich M, et al. Hip Joint Contact Pressure Distribution During Pavlik Harness Treatment of an Infant Hip: A Patient-Specific Finite Element Model. J Biomech Eng, 2018;140(7). 10.1115/1.4039827. 10.1115/1.4039827 29715363

[29] Zou Z, Chávez-Arreola A, Mandal P (2013). Optimization of the position of the acetabulum in a ganz periacetabular osteotomy by finite element analysis. J Orthop Res..

[30] Sarikaya B, Sipahioglu S, Sarikaya ZB (2018). The early radiological effects of Dega and Pemberton osteotomies on hip development in children aged 4–8 years with developmental dysplasia of the hip. J Pediatr Orthop B.

[31] Ming-Hua D, Rui-Jiang X, Wen-Chao L (2016). The high osteotomy cut of Dega procedure for developmental dysplasia of the hip in children under 6 years of age. Der Orthopde.

[32] Jinghang Yu, Shuang L, Lianyong Li (2017). Correlation of the pathologic morphology on MRI with International Hip Dysplasia Institute classification in developmental dysplasia of the hip. Chin J Tissue Eng Res.

[33] Li Y, Guo Y, Li M (2018). Acetabular index is the best predictor of late residual acetabular dysplasia after closed reduction in developmental dysplasia of the hip. Int Orthop.

[34] Issn A, Ner A, Kokara N (2016). Comparison of open reduction alone and open reduction plus Dega osteotomy in developmental dysplasia of the hip. J Pediatr Orthop B.

[35] Shefelbine SJ, Carter DR (2004). Mechanobiological predictions of growth front morphology in developmental hip dysplasia. J Orthop Res.

[36] Yadav P, Shefelbine S, Gutierrez-Farewik E (2016). Effect of growth plate geometry and growth direction on prediction of proximal femoral morphology. J Biomech.

[37] Vafaeian B, Zonoobi D, Mabee M (2017). Finite element analysis of mechanical behavior of human dysplastic hip joints: a systematic review. Osteoarthritis Cartilage.

[38] Chegini S, Beck M, Ferguson SJ (2010). The effects of impingement and dysplasia on stress distributions in the hip joint during sitting and walking: A finite element analysis. J Orthop Res.

[39] Senaran H, Bowen JR, Harcke HT (2007). Avascular necrosis rate in early reduction after failed Pavlik harness treatment of developmental dysplasia of the hip. J Pediatr Orthop.

[40] Tönnis D (1987). Ischemic Necrosis of the Femoral Head in the Treatment of Congenital Hip Dislocation [M].

[41] Bae JY, Kwak DS, Park KS (2013). Finite element analysis of the multiple drilling technique for early osteonecrosis of the femoral head. Ann Biomed Eng.

[42] Zhang Y, Tian K, Ma X (2018). Analysis of damage in relation to different classifications of pre-collapse osteonecrosis of the femoral head. J Int Med Res.

[43] Chen L, Hong G, Fang B (2017). Predicting the collapse of the femoral head due to osteonecrosis: From basic methods to application prospects. J Orthop Transl..

[44] Wen P, Zhang Y, Hao L (2020). The effect of the necrotic area on the biomechanics of the femoral head - a finite element study. BMC Musculoskelet Disord.

[45] Wen PF, Guo WS, Zhang QD (2017). Significance of Lateral Pillar in Osteonecrosis of Femoral Head: A Finite Element Analysis. Chin Med J (Engl).

[46] Russell ME, Shivanna KH, Grosland NM (2006). Cartilage contact pressure elevations in dysplastic hips: a chronic overload model. J Orthop Surg Res.

[47] Tibor LM, Sink EL (2012). Periacetabular Osteotomy for Hip Preservation. Orthop Clin North Am.

[48] Domb B, Lareau J, Redmond JM (2014). Combined Hip Arthroscopy and Periacetabular Osteotomy: Indications, Advantages, Technique, and Complications. Arthrosc Tech.

[49] Abraham CL, Knight SJ, Peters CL (2017). Patient-specific chondrolabral contact mechanics in patients with acetabular dysplasia following treatment with peri-acetabular osteotomy. Osteoarthr Cartil.

[50] Knight SJ, Abraham CL, Peters CL (2017). Changes in chondrolabral mechanics, coverage, and congruency following peri-acetabular osteotomy for treatment of acetabular retroversion: A patient-specific finite element study. J Orthop Res.

